# Differential gene expression and potential regulatory network of fatty acid biosynthesis during fruit and leaf development in yellowhorn (*Xanthoceras sorbifolium*), an oil-producing tree with significant deployment values

**DOI:** 10.3389/fpls.2023.1297817

**Published:** 2024-01-19

**Authors:** Tian-Le Shi, Hai-Yao Ma, Xinrui Wang, Hui Liu, Xue-Mei Yan, Xue-Chan Tian, Zhi-Chao Li, Yu-Tao Bao, Zhao-Yang Chen, Shi-Wei Zhao, Qiuhong Xiang, Kai-Hua Jia, Shuai Nie, Wenbin Guan, Jian-Feng Mao

**Affiliations:** ^1^ State Key Laboratory of Tree Genetics and Breeding, Ministry of Education, School of Ecology and Nature Conservation, College of Biological Sciences and Technology, Beijing Forestry University, Beijing, China; ^2^ Key Laboratory of Genetics and Breeding in Forest Trees and Ornamental Plants, Ministry of Education, School of Ecology and Nature Conservation, College of Biological Sciences and Technology, Beijing Forestry University, Beijing, China; ^3^ Institute of Crop Germplasm Resources, Shandong Academy of Agricultural Sciences, Ji’nan, China; ^4^ Guangdong Academy of Agricultural Sciences & Key Laboratory of Genetics and Breeding of High-Quality Rice in Southern China (Co-construction by Ministry and Province), Rice Research Institute, Guangzhou, China; ^5^ Ministry of Agriculture and Rural Affairs & Guangdong Key Laboratory of New Technology in Rice Breeding, Rice Research Institute, Guangzhou, China; ^6^ Department of Plant Physiology, Umeå Plant Science Centre, Umeå University, Umeå, Sweden

**Keywords:** fatty acid biosynthesis, time-ordered gene co-expression network (TO-GCN), oleosins, fruit and leaf development, yellowhorn

## Abstract

*Xanthoceras sorbifolium* (yellowhorn) is a woody oil plant with super stress resistance and excellent oil characteristics. The yellowhorn oil can be used as biofuel and edible oil with high nutritional and medicinal value. However, genetic studies on yellowhorn are just in the beginning, and fundamental biological questions regarding its very long-chain fatty acid (VLCFA) biosynthesis pathway remain largely unknown. In this study, we reconstructed the VLCFA biosynthesis pathway and annotated 137 genes encoding relevant enzymes. We identified four oleosin genes that package triacylglycerols (TAGs) and are specifically expressed in fruits, likely playing key roles in yellowhorn oil production. Especially, by examining time-ordered gene co-expression network (TO-GCN) constructed from fruit and leaf developments, we identified key enzymatic genes and potential regulatory transcription factors involved in VLCFA synthesis. In fruits, we further inferred a hierarchical regulatory network with MYB-related (*XS03G0296800*) and B3 (*XS02G0057600*) transcription factors as top-tier regulators, providing clues into factors controlling carbon flux into fatty acids. Our results offer new insights into key genes and transcriptional regulators governing fatty acid production in yellowhorn, laying the foundation for efforts to optimize oil content and fatty acid composition. Moreover, the gene expression patterns and putative regulatory relationships identified here will inform metabolic engineering and molecular breeding approaches tailored to meet biofuel and bioproduct demands.

## Introduction

Fatty acids, the major lipids in plants, play a crucial role in plant growth, development, and stress response, as it is involved in the production of lipids, which are essential components of cellular membranes, energy storage, and signaling molecules (Joubès et al., 2008; Kim, 2020). Fatty acids and various of lipids are important sources of energy in diet and sources of essential and fatty acid and fat-soluble vitamins. Identifying the key genes in fatty acids biosynthesis and resolving the relevant gene regulatory circuit are crucial for fatty acid production and genetic improvement of oil-producing plant.

According to carbon chain length, fatty acids can be classified into short-chain, medium-chain, long-chain and VLCFAs (very long-chain fatty acids). Among these fatty acids, VLCFAs, as a unique class of fatty acids, have extremely important biological functions and application values. VLCFAs are essential constituents of eukaryotic organisms. The variation in carbon chain length and degree of unsaturation of VLCFAs is the basis for their structural and functional diversity. VLCFAs mainly have four forms: triacylglycerol (TAG), wax, phosphoglyceride, and sphingolipid (Bach & Faure, 2010). They are crucial for the growth and development of organisms, as well as for responses to biotic and abiotic stresses. VLCFAs are important for various biological processes, such as storage lipids in seed, cuticular waxes, and suberin polymers. Elucidating the genes and enzymes associated with fatty acid elongation is key to understanding VLCFA metabolic pathway in oil-rich plants (Joubès et al., 2008; Bach & Faure, 2010).

In higher plants, the synthesis of VLCFAs can be divided into two main phases: *de novo* fatty acid biosynthesis and fatty acid elongation (Rajasekharan & Nachiappan, 2010). The initial reactions of fatty acid biosynthesis occur in the plastids and use acetyl-CoA as a precursor (Ohlrogge and Browse, 1995). Acetyl-CoA carboxylase (ACCase) catalyzes the first committed step, the irreversible carboxylation of acetyl-CoA to form malonyl-CoA (Nikolau et al., 2003). Next, the fatty acid synthase (FAS) complex consisting of Ketoacyl-ACP synthase (KAS), Ketoacyl-ACP reductas (KAR), Hydroxyacyl-ACP dehydratase (HAD), and Enoyl-ACP reductase (ENR) iteratively condensates malonyl-CoA with an acyl acceptor to produce C16-C18 fatty acids, primarily palmitic acid (C16:0), stearic acid (C18:0), and oleic acid (C18:1) (Lee et al., 2020). Further desaturation and elongation produce longer and more unsaturated fatty acids. The endoplasmic reticulum membrane-bound Δ12-oleic acid desaturase (FAD2) converts oleic acid to linoleic acid (C18:2) by adding a second double bond. VLCFAs are formed by the endoplasmic reticulum-localized fatty acid elongase (FAE) consisting of ketoacyl-CoA synthase (KCS), ketoacyl-CoA reductase (KCR), hydroxy acyl-CoA dehydratase (HCD), and enoyl-CoA reductase (ECR) which sequentially adds two carbon units, using malonyl-CoA as the donor (Haslam and Kunst, 2013). For VLCFAs (>C20), additional elongations are catalyzed by enzymes such as β-ketoacyl-CoA synthases (KCS) (Joubès et al., 2008). Different types of VLCFAs together with other compounds create a surface barrier on leaves, stems and roots. They are also part of the storage lipids in the seeds of some plant species. Between the endoplasmic reticulum membranes of seed cells, triacylglycerol (TAG) is synthesized and stored in oil body.

Several woody plants are known for their high oil content, such as *Camellia oleifera* (tea-oil tree) (Ye et al., 2021), *Juglans regia* (Walnut) (Huang et al., 2021) and *Xanthoceras sorbifolium* (yellowhorn) (Liu et al., 2021). In these species, the main oil-producing tissues are typically found in their fruits. Oleosin, a protein encoded by the *Oleosin* gene, plays a key role in stabilizing the oil body and regulating seed oil content (Yuan et al., 2021). These lipids are usually stored as triacylglycerol (TAG), the end product of very long-chain fatty acids (Siloto et al., 2006). Oleosins can be divided into five lineages in green plants: (1) the primitive (P) oleosins found in green algae and primitive plants, such as mosses and ferns; (2) the universal (U) oleosins found in all land plants; (3) the fruit low molecular-weight (SL) oleosins found in the fruits of angiosperms and gymnosperms; (4) the fruit high molecular-weight (SH) oleosins found in the fruits of angiosperms; (5) the tapetum (T) oleosins found in the tapetum of Brassicaceae species (Huang, 2018). Moreover, the overexpression of certain *Oleosin* genes has been shown to increase oil content and alter fatty acid composition in transgenic plants (Miquel et al., 2014; Zhang et al., 2019). Therefore, characterizing *Oleosin* gene family is relevant for illuminating VLCFA biosynthesis pathway.

Yellowhorn (*Xanthoceras sorbifolium*, Sapindaceae) is a woody oilseed tree species endemic to northern China with a high oil content. Yellowhorn seeds have a high oil content of up to 68%, which is higher than walnut oil, tea oil, and olive oil (Yao et al., 2013; Venegas-Calerón et al., 2017; Yu et al., 2017). The proportion of unsaturated fatty acids (UFAs) in the oil is as high as 90.9% (Yao et al., 2013). Yellowhorn oil can be used as a biofuel and is also edible, making it highly valuable for both consumption and medicinal purposes. Notably, nervonic acid (cis-15-tetracosenoic acid), a very long-chain fatty acid, is relatively rare in plants but accounts for 1.5-3.0% of yellowhorn seed oil (Ruan et al., 2017). Nervonic acid is an essential component of myelin phospholipid biosynthesis in the central and peripheral nervous systems and plays a special role in promoting the repair and regeneration of nerve fibers in damaged brain tissue (Oda et al., 2005; Amminger et al., 2012). Increasing the nervonic acid content in seeds will become an important breeding goal for yellowhorn.

Although the fatty acid synthesis pathway of *Xanthoceras sorbifolia* was relatively well-studied, previous studies had mainly focused on a certain class of important enzymes, such as KCS. Few investigations as to the dynamic molecular changes in a network about fatty acids synthesis of yellowhorn, and no comparative study investigated the difference among multiple tissues. Time-ordered gene co-expression network (TO-GCN) analysis coupled with landscape transcriptomic characterization provides powerful tools (Chang et al., 2019). The TO-GCN approach can elucidate complex biosynthetic pathways in plants. For example, it has been used to predict genes involved in flower color determination in azalea (*Rhododendron simsii*) (Yang et al., 2020), to study the response of *Pinus tabuliformis* to UV-B and UV-C radiation (Xu et al., 2022), and to investigate salt tolerance mechanisms in poplar “84K” (Zhao et al., 2023). This approach can provide insights into the coordinated expression of genes encoding enzymes and transcription factors (TFs) that regulate fatty acid synthesis, as well as the potential regulatory elements that control their expression.

In this study, we first reconstructed the whole pathway of VCLFA biosynthesis and annotated the genes encoding related enzymes in the pathway. Key enzymatic genes and potential regulatory TFs of VCLFA synthesis were identified by examining the TO-GCN networks developed from fruit and leaf development. We also identified four *Oleosin* genes encapsulating triacylglycerols (TAGs), which are specifically expressed in the fruit, and may be key to fruit oil production in yellowhorn. Identification of candidate key enzymatic and regulatory genes related to fatty acid elongation and oil body biogenesis will inform biotechnology and molecular breeding in yellowhorn to furth optimize VLCFA-derived fuel production in this emerging woody bioenergy plant.

## Materials and methods

### Plant materials and sequencing

**Figure 1:**
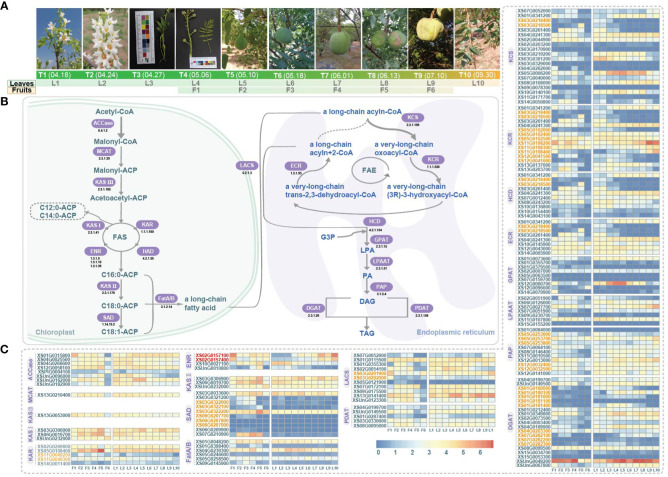
Sampling of fruit and leaf development and gene expression profile related to fatty acid synthesis pathway in yellowhorn. **(A)** Six stages of fruit development: F1-F6; Ten stages of leaf development: L1-L10. T1-T10 are denoting the date and the phenotypes of the sampled stages. **(B)** The fatty acid synthesis pathway. Key enzymes are highlighted, ACCase, Acetyl-CoA carboxylase (EC 6.4.1.2); MCAT, Malonyl-CoA acyl carrier protein transferase (EC 2.3.1.39); KAS III, Ketoacyl-ACP synthase III (EC 2.3.1.180); KAS I, Ketoacyl-ACP synthase I (EC 2.3.1.41); KAR, Ketoacyl-ACP reductase (EC 1.1.1.100); HAD, Hydroxyacyl-ACP dehydratase (EC 4.2.1.59); ENR, Enoyl-ACP reductase (EC 1.3.1.9/1.3.1.10/1.3.1.39); KAS II, Ketoacyl-ACP synthase II (EC 2.3.1.179); SAD, Stearoyl-ACP desaturase (EC 1.14.19.2); Fat, Fatty acyl-ACP thioesterase (EC 3.1.2.14); LACS, Long-chain acyl-CoA synthetase, (EC 6.2.1.3); KCS, 3-ketoacyl-CoA synthase (EC 2.3.1.199); KCR, Ketoacyl-CoA reductase (EC 1.1.1.330); HCD, 3-hydroxy acyl-CoA dehydratase (EC 4.2.1.134); ECR, Enoyl-CoA reductase (EC 1.3.1.93); FAE, Fatty acid elongase; GPAT, Glycerol-3-phosphate acyltransferases (EC 2.3.1.15); LPAAT, Lysophosphatidic acid acyltransferase (EC 2.3.1.51); PAP, Phosphatidic acid phosphatise (EC 3.1.3.4); DGAT, Diacylglyceol acyltransferase (EC 2.3.1.20); PDAT, Phospholipids, diacylglycerol acyltransferase (EC 2.3.1.158); G3P, Glycerol-3-Phosphate; LPA, Lysophosphatidic acid; PA, Phosphatidic acid; DAG, Diacylglycerol; TAG, Triacylglycerol. **(C)** Gene expression profiles (in normalized TPMs) at different time points for fruits (F1-F6, from left to right in each heatmap) and leaves (L1-L10). Gene names in orange denote genes of tandem duplication. Red gene names denoted genes of proximal duplication.

In this study, we collected 12 and 37 transcriptome samples from different developmental stages of the fruit (six stages based on sampling dates, F1-F6) and leaf (ten stages based on sampling dates, L1-L10) in yellowhorn, respectively (**Figure 1A**; **Supplementary Tables 2**, **3**). The samples were obtained from a yellowhorn stand located in Dadongliu Forest Farm, Changping District, Beijing, China. RNA extraction was performed using the QIAGEN RNeasy Plant Mini Kit (QIAGEN, USA). The extracted RNA was then converted into cDNA, and RNA-Seq libraries were constructed using the mRNASeq Sample Preparation Kit (Illumina Inc., San Diego, CA, USA). Subsequently, paired-end sequencing was conducted on the Illumina HiSeq X Ten platform.

### Transcriptome assembly and gene expression quantification

Raw reads from Illumina sequencing were filtered to obtain clean reads by removing low-quality read ends (quality < 30), adapter sequences, reads with ambiguous bases N, and reads shorter than 60 bp (Kechin et al., 2017). And then clean reads were mapped to the reference genome using HiSat2 v2.1.0 (Kim et al., 2015) with the parameter “-k 1.” We calculated the Transcripts per Kilobase Million (TPM) values of genes using StringTie v1.3.5 (Pertea et al., 2015). Differentially expressed genes (DEGs) were identified using DESeq2 package v1.34.0 (Love et al., 2014), with 0.05 as the FDR cut-off and a log2 fold change (FC) cut-off of 1. The whole genome assembly of one yellowhorn accession, “Jinguanxiapei” (“JGXP”) (Liu et al., 2021) was used as the reference genome.

### Very long-chain fatty acid biosynthesis pathway

The protein-coding gene models predicted from the whole genome of one yellowhorn accession, “Jinguanxiapei” (“JGXP”) (Liu et al., 2021) were further annotated according to their enzyme function classes using E2P2 (Ensemble Enzyme Prediction Pipeline) v3.1 (Chae et al., 2014). Subsequently, annotated genes were assigned to specific biosynthesis pathway as it is illustrated in the PLANTCYC v13.03 database using Pathway Tools v22.5 (Karp et al., 2016), thus we gained the gene annotation of the VLCFA biosynthesis in yellowhorn.

### Chromosomal location and gene duplication of enzymatic genes related to fatty acid synthesis

The analysis of gene duplication and the chromosomal location of genes can enhance our understanding of duplication and regulation mechanism of the enzymatic genes. In the case of yellowhorn, we obtained information of the chromosomal location of each enzymatic gene from the yellowhorn gene annotation (Liu et al., 2021). Subsequently, TBtools v0.66836 (Chen et al., 2020) was utilized to map and visualize the chromosome distribution.

### Genome−wide identification and characterization of *Oleosin* gene family

To identify potential members of the *Oleosin* gene family in yellowhorn, we downloaded the Hidden Markov Model (HMM) profile of the oleosin domain (PF01277) from Pfam (http://pfam.xfam.org/). This HMM profile was used as query to search protein sequence databases using the HMMER software (http://hmmer.org/) with an E-value threshold of 1e-10. The obtained protein sequences were manually inspected using SMART (http://smart.embl-heidelberg.de/) to verify the presence of conserved domains. Protein sequences that lacked conserved domains were excluded from further analysis. Additionally, the presence of target domain was confirmed using the NCBI Batch Web CD-Search Tool (https://www.ncbi.nlm.nih.gov/Structure/bwrpsb/bwrpsb.cgi) with default parameters.

In addition, understanding gene function often relies on information regarding gene structure, conserved motifs, and cis-elements. The MEME Suite online tool (http://meme-suite.org/meme/) was utilized to predict the conserved motifs of the potential *Oleosin* genes. Further annotation of the identified motifs was performed using the NCBI Web CD-Search Tool. Visualization of the conserved motifs and gene structure was accomplished by using TBtools.

### Gene family construction

In order to accurately classify the 16 *Oleosin* genes from *Arabidopsis thaliana* (Kim et al., 2002) into subfamilies, a phylogenetic tree was constructed using the gene members identified from *A. thaliana*, as well as *Oleosin* genes from yellowhorn and date palm. The construction process involved several steps. First, the protein sequences were aligned using MUSCLE v3.8.425 (Edgar, 2004) with default parameters, and low-quality regions were filtered using trimAl v1.4.rev15 (Capella-Gutierrez et al., 2009). The filtered alignment sequences were then used to build a maximum likelihood phylogenetic tree by using IQ-TREE v1.6.7 (Nguyen et al., 2015), with the best-fit model determined by ModelFinder (Kalyaanamoorthy et al., 2017) as WAG+F+I+G4. To assess the reliability of the tree branches, 1,000 bootstrap replicates were performed. The subgroup assignment of *Oleosin* family members was based on the treatment established in a previous study (Huang, 2018).

### Identification of transcription factors and transcription factor binding site of yellowhorn

Genes annotated in the yellowhorn genome (Liu et al., 2021) were analyzed using the PlantRegMap database (Jin et al., 2015) to identify TFs showing homology to *A. thaliana*. A total of 1,684 TF genes were identified in the genome (**Supplementary Table 10**). The identification of TF binding sites is crucial for understanding transcriptional regulation. In this study, we employed the PlantRegMap platform (Tian et al., 2020) to predict TF binding sites within the promoter regions (2,000 bp upstream of the genes) of candidate genes involved in the fatty acid biosynthesis pathway.

### Gene functional enrichment

We used hypergeometric tests to assess whether particular functional categories from Gene Ontology (GO) were significantly enriched in the set of yellowhorn genes within the genome. Functional enrichment analysis was conducted using clusterProfiler v3.8.1 (Yu et al., 2012), considering all annotated yellowhorn genes in this study as the background set.

### Co-expression network construction

We use a recently developed method to reconstruct time-ordered gene co-expression networks (TO-GCNs) (Chang et al., 2019). To generate these networks, we utilized samples obtained from five distinct developmental stages of the fruit (F1-F5) and eight different developmental stages of the leaf (L1-L8). Firstly, we performed differential expression analysis between any two time points using the DEseq2 package (Love et al., 2014), with a log2 fold-change (FC) cutoff value of 1 and p.adjust < 0.05. Differentially expressed genes (DEGs) with average TPM expression values less than 0.5 at each time point were excluded. For the TO-GCNs developed from fruit and leaf samples, we applied threshold values of 0.8 and 0.85 as the thresholds of Pearson correlation between TFs and structural genes for network construction, respectively.

## Results

### Differential expression of genes involved in fatty acid biosynthesis during fruit and leaf development

The morphological characteristics of six different stages (F1 to F6) of fruit development and ten different stages (L1 to L10) of leaf development were shown in **Figure 1A**. We reconstructed the pathway responsible for fatty acid biosynthesis in yellowhorn, and a total of 137 genes encoding related enzymes were annotated (**Figure 1B, C**; **Supplementary Table 1**). During the phase of *de novo* fatty acid biosynthesis, three, five and four genes encoding KAS I, KAR and ENR, respectively, were annotated to form the FAS complex. However, the gene encoding HAD was not annotated. The genes encoding KAS I (*XS03G0306900*, *XS06G0019700* and *XSUnG0232000*) and genes encoding KAR (*XS02G0269800* and *XS05G0108400*) maintained consistent high expression throughout fruit and leaf development. Conversely, the genes encoding ENR exhibited varied expression patterns. During the phase of fatty acid elongation, 20, 15, 10 and 8 genes encoding KCS, KCR, HCD and ECR, respectively, were annotated to form the FAE complex. Among them, the gene encoding KCS (*XS02G0044900*) and genes encoding KCR (*XS05G0102500*, *XS11G0198200* and *XS11G0198300*) were consistently highly expressed throughout the developmental stages of fruit and leaf.

Most enzymatic genes showed coordinated expression in fruits and leaves. For example, one gene *XSPtG0004100* encoding the rate-limiting enzyme ACCase showed low expression levels, while seven other ACCase genes exhibited higher expression levels in all studied stages of fruit and leaf development (**Figure 1C**). We also observed that a few genes showed tissue-specific expression, such as the gene *XS07G0210900* encoding SAD was highly expressed during the F2-F5 stages of fruit development but showed negligible expression in leaf development (**Figure 1C**). Additionally, in fruit, most genes were found activated in F2-F5 but suppressed in F1 and F6, reflecting possibly slow-fast-slow developmental transitions in yellowhorn (**Figure 1C**). In leaves, enzymatic genes related to VLCFA biosynthesis exhibited diverse stage-preferences, with some were induced only in the early or late stages (**Figure 1C**). Some enzymatic genes, such as genes *XSUnG0096000* and *XS02G0157100*, displayed high expression exclusively during early or late stages of development. Additionally, some enzymatic genes showed neither no expression nor low expression levels in both fruit and leaf, suggesting that they might be pseudogenes. Some genes with sustained high expression levels were considered crucial in fatty acid biosynthesis, such as *XS13G0141400* encoding LACS, *XS02G0044900* encoding KCS, *XS11G0198200* encoding KCR and *XSUnG0049200* encoding. These key genes encoding critical enzymes in fatty acid biosynthesis provided promising targets for molecular breeding and metabolic engineering efforts aimed at optimizing and enhancing fatty acid biosynthesis in yellowhorn.

### Chromosomal localization of enzymatic genes and biosynthetic gene clusters related to fatty acid biosynthesis

**Figure 2:**
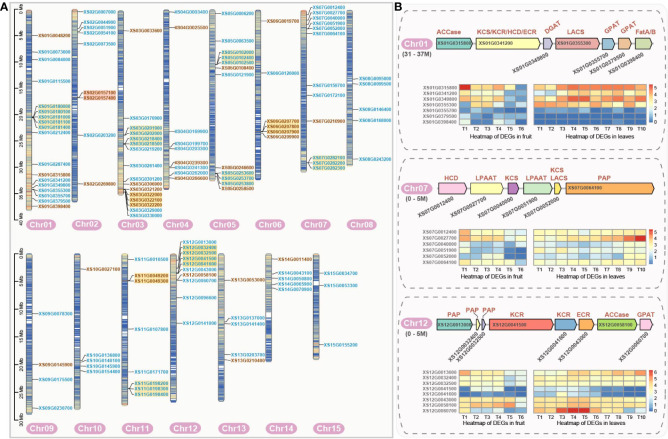
Chromosomal localization of genes encoding fatty acid biosynthesis-related enzymes and the gene clusters in yellowhorn. **(A)** Chromosomal localization of genes encoding fatty acid synthesis-related enzymes. Gene names in blue indicate enzymatic genes in the phase of fatty acid *de novo* biosynthesis. Gene names in brown represent genes encoding enzymes in the phase of fatty acid elongation. Enzymatic gene names highlighted with yellow background denote tandem duplication genes. Enzyme genes with orange background denote proximal duplication genes. **(B)** Gene clusters related to fatty acid biosynthesis and their expression profile. Gene expression profiles (in normalized TPMs) of enzymatic genes are presented in the heatmap alongside with the gene names. The bar represents the expression level of each gene (z-score). Low to high expression is indicated by a change in color from blue to red. Gene (*XS01G0341200*) was simultaneously annotated as the KCS, KCR, ECR and HCD enzymatic genes as it is containing four conserved protein domains by E2P2 (Ensemble Enzyme Prediction Pipeline) software. Gene (*XS07G0052000*) was simultaneously annotated as the KCS, and LACS by E2P2.

The analysis of gene duplication and the chromosomal location of genes can enhance our understanding of duplication and regulation mechanism of the enzymatic genes. A total of 127 enzymatic genes related to fatty acid biosynthesis were mapped to 15 chromosomes (**Figure 2A**). Additionally, 10 genes were localized to the chloroplast and unanchored contigs. It is worth noting that these genes are not uniformly distributed across all chromosomes (**Figure 2A**). Chr01 was found to carry the highest number of genes (18), whereas Chr15 contained only three genes. Chr09 contained 4 genes, just one more than the shortest chromosome (Chr15). The number of genes on other chromosomes ranged from 5 to 16. Therefore, no clear correlation was observed between the length of the chromosome and the number of enzymatic genes it carries. Interestingly, we also identified a significant number of tandem duplications within these genes, which may share transcriptional regulatory elements to enhance the efficiency of metabolic pathways (**Figure 2A**).

Moreover, three significant gene clusters, associated with the VLCFA biosynthesis, were found on Chr01 (31-37M), Chr07 (0-5M), and Chr12 (0-5M) (**Figure 2B**). Notably, the genes within the cluster on Chr07 were crucial enzymatic genes required for VLCFA biosynthesis. The gene (*XS07G0027700*) encoding LPAAT generally showed high expression levels in both fruit and leaf, but the expression level was higher in the first and middle stages of fruit, and also in the middle and late stages of leaf. The genes in the clusters found on Chr01 and Chr12 encoded enzymes spanned the entire process of fatty acid biosynthesis. The gene clusters encoded included the rate-limiting enzyme ACCase (*XS01G0315800* and *XS12G0058100*) in the *de novo* fatty acid biosynthesis, which is consistently highly expressed throughout fruit and leaf development. Also, the genes encoding LACS (*XS01G0355300*), KCS (*XS01G0341200*), ECR (*XS12G0043000*) and PAP (*XS12G0013000*, *XS12G0032400* and *XS12G0032500*) in the fatty acid elongation were expressed at high levels from early to middle stages and then decreased at the F6 stage in fruit, however, this pattern was less evident in the leaf.

### Phylogenetic relationships of *Oleosin* genes

**Figure 3:**
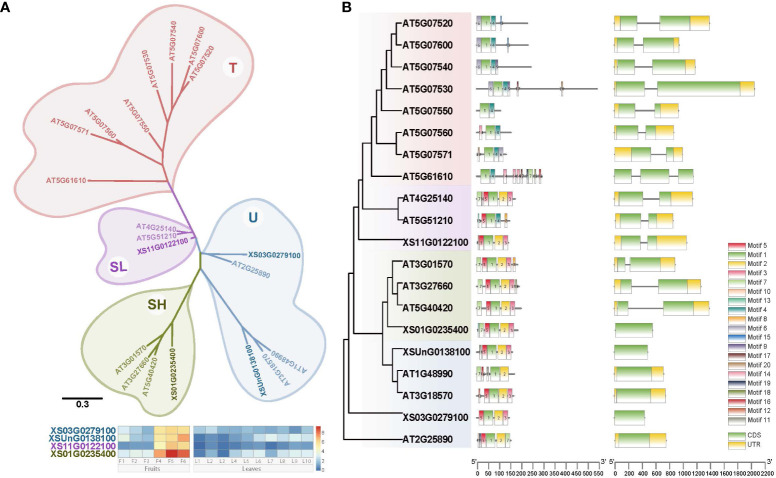
Phylogenetic tree and gene structure of *Oleosin* genes from yellowhorn and Arabidopsis. **(A)** Phylogenetic reconstruction for *Oleosin* genes from yellowhorn and Arabidopsis. Gene expression profiles (in normalized TPMs) of *Oleosin* genes in yellowhorn are presented in the heatmap alongside with the gene names. The bar represents the expression level of each gene (z-score). Low to high expression is indicated by a change in color from blue to red. **(B)** Gene structure of oleosins. Twenty conserved motifs indicated by different colors. Gene structure with exons and introns indicated.

Oleosins are plant proteins primarily found in oil bodies, playing a crucial role in their stabilization and biogenesis, and are classified into four subfamilies: U, SL, SH, and T. Each subfamily is distinguished by differences in their central hydrophobic domain and variable N- and C-terminal regions, creating diversity in their functionalities and implications in areas such as seed development, lipid metabolism, and stress response in different plant species. To investigate the dynamic expression of the *Oleosin* gene family for the VLCFA biosynthesis, we identified in total four *Oleosin* genes in yellowhorn by homology searching for conserved domains (PF00135) (**Figure 3A**). Based on the phylogenetic tree, the 20 *Oleosin* genes (four from yellowhorn, 16 from *A. thaliana*) were assigned into four clades: U, SL, SH, and T, with T being specific to *A. thaliana* (**Figure 3A**). Each SL and SH evolutionary clade contained one *Oleosin* gene from yellowhorn, while the U clade contained two members. In addition, it was very clear that the four *Oleosin* genes in yellowhorn were distinctly tissue-specific in the fruit. They were highly expressed in the middle and late stages of fruit development (F4 - F6) and hardly expressed in the leaf.

Gene structure and motif conservation were critical factors in understanding the evolutionary dynamics and functional diversification of gene families. The majority of *A. thaliana Oleosin* genes (12 out of 16, accounting for 75%) contained one intron, but 3 of 4 genes in yellowhorn were intronless (**Figure 3B**). In clade U, none of the *Oleosin* genes in both yellowhorn and *A. thaliana* had introns. The SL and SH *Oleosin* genes in *A. thaliana* both had one intron, whereas in yellowhorn, the SL *Oleosin* gene (*XS11G0122100*) contained an intron and the SH *Oleosin* gene (*XS01G0235400*) lacks intron (**Figure 3B**). Furthermore, we predicted the conserved motifs of oleosins and discovered that conserved motifs distinguished the clades. All genes had Motif 1, which belongs to the oleosin domain. Conserved motifs in the SL, SH, and U clades were generally similar, primarily including motifs 1, 2, 3, and 5, with motifs 2, 3, and 5 being unique to these three clades. However, the T clade in *A. thaliana* exhibited distinct motif conservation, characterized by different motifs compared to the other three clades, particularly motifs 1, 4, and 6.

### Transcriptomic differences in fruit and leaf development

**Figure 4:**
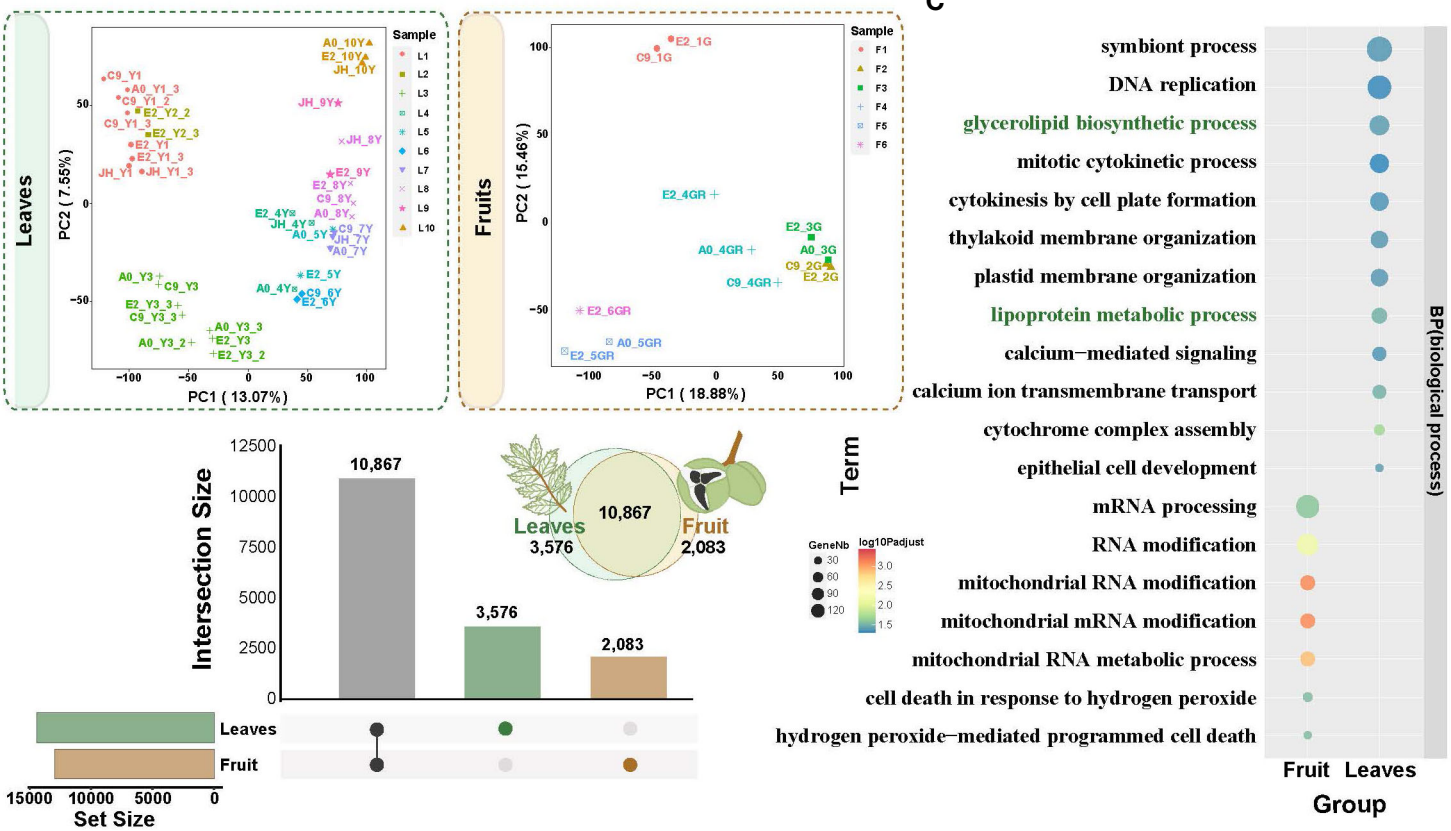
Principal component analysis (PCA) of transcriptomes from fruit and leaf development and the specific expression. **(A)** PCA gene expression in yellowhorn fruit and leaf development. **(B)** Upset plot of differentially expressed genes between fruit and leaf development. Brown columns indicate differentially expressed genes specific to the fruits. Green columns indicate differentially expressed genes specific to the leaves. **(C)** Functional enrichment analysis of specifically expressed genes in fruit and leaves. The enriched GO terms with corrected *P* value < 0.05 are presented. The color of circles denotes the statistical significance of the enriched GO terms. The size of the circles denotes the number of genes in a GO term. ‘P adjust’ is the Benjamini-Hochberg false discovery rate (FDR) adjusted *P* value.

To investigate the regulatory genes potentially involved in fatty acid biosynthesis in the fruit and leaf development, we examined the transcriptomes of developmental stages of the fruit (12 samples from 6 developmental stages, F1-F6) and leaf (37 samples from 10 developmental stages, L1-L10) (**Figure 1A**; **Supplementary Tables 2**, **3**). PCA (Principal Component Analysis) clearly separated the different developmental stages of the fruit and leaf tissues (**Figure 4A**; **Supplementary Tables 2**, **3**). The biological replicates within each group were located closely together, confirming the consistency of the sampling and experimental procedures in our study. Also, it was worth noting that the samples of leaves were arranged clearly in order of development stages (from left to right, **Figure 4B**), suggesting a time-ordered gene regulation and coordinated gene expression along the leaf development.

A total of 12,950 and 14,443 differentially expressed genes (DEGs) were identified in the fruit and leaf tissues, respectively (**Supplementary Figures 1**, **2**). Among these DEGs, 2,083 were specifically expressed in the fruit, while 3,576 were specifically expressed in the leaf (**Figure 4B**). Gene Ontology (GO) enrichment analysis for these specific DEGs was conducted (**Figure 4C**; **Supplementary Figure 3**). In fruit, the enriched processes were primarily associated with molecular modifications and cell in response to hydrogen peroxide (**Supplementary Table 4**). And the specific DEGs were enriched in processes related to glycerolipid biosynthetic process and lipoprotein metabolic process in leaf (**Supplementary Table 5**). This suggested the presence of fatty acid biosynthesis processes in leaves, such as those related to the formation of cuticle in the epidermis of yellowhorn leaves, which serves as a defense mechanism against biotic and abiotic stresses (**Figure 4C**).

### Time-ordered gene co-expression network related to fatty acid biosynthesis

We performed a time-dependent comparative transcriptomic analysis to investigate the gene expression pattern during fruit and leaf development in yellowhorn. A total of 10,111 DEGs were identified (including 601 TFs and 9,510 non-TFs) between any two time points (**Supplementary Table 6**). Similarly, for leaf development, we detected 8,445 DEGs (629 TFs and 7,816 non-TFs) across nine time points (L1-L9) (**Supplementary Table 7**). In fruits and leaves, the TFs *XS01G0336800* (lateral organ boundaries domain, LBD) (**Supplementary Figure 4**) and *XS03G0212300* (cysteine-rich polycomb-like protein, CPP) (**Supplementary Figure 5**) were identified as the initial nodes, which were further used as seeds for constructing the time-ordered gene co-expression networks (TO-GCNs), respectively (**Supplementary Figures 6**, **7**). These TFs exhibited high expression levels at the first time point and gradually decreased at subsequent time points. These 6 and 9 levels (subnetworks) matched the expression peaks of DEGs at five of the fruit and eight of the leaf developmental stages, as shown by the red squares (high expression levels) along the diagonal in the heat-map, which formed the basis for the inference of gene regulatory relationships (**Supplementary Figures 8**, **9**).

**Figure 5:**
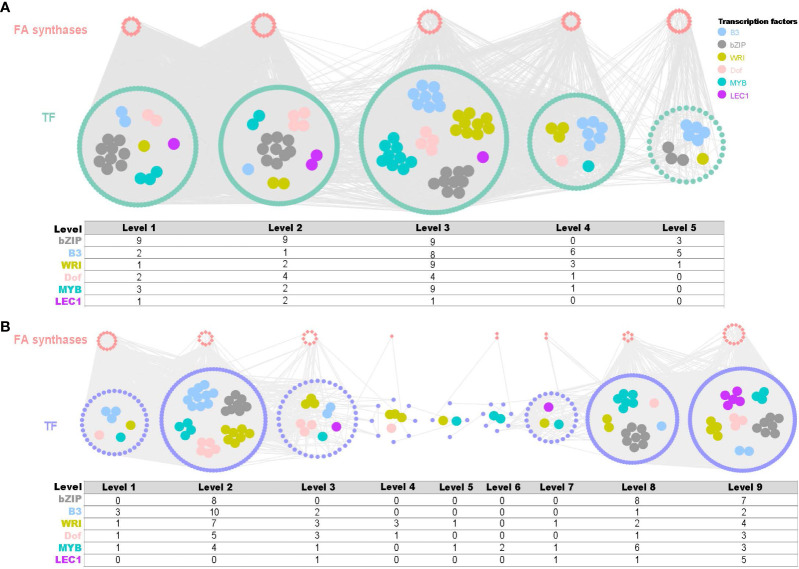
Time-ordered gene co-expression network (TO-GCN) associated with fatty acid synthesis in fruit and leaf development. **(A)** Predicted regulatory network and the connection among TFs and structural genes (enzymatic genes) involved in fatty acid biosynthesis pathway of fruits and statistics on the number of important transcription factors. Inside the green circles, wathet blue nodes represent *B3* genes, grey nodes represent *bZIP* genes, yellow nodes represent *WRI* genes, pink nodes represent *Dof* genes, turquoise nodes represent *MYB* genes, and purple nodes represent *LEC1* genes. Green nodes represent remaining TFs. Outside green circles, points represent the enzymatic genes of the fatty acid biosynthesis pathway, locating on the top. Level 1 to Level 5 indicate the levels identified in the f-ordered gene co-expression network. **(B)** Predicted regulatory network and the connection among TFs and structural genes involved in fatty acid biosynthesis pathway of leaves statistics on the number of important transcription factors. Inside the purple circles, wathet blue nodes represent *B3* genes, grey nodes represent *bZIP* genes, yellow nodes represent *WRI* genes, pink nodes represent *Dof* genes, turquoise nodes represent *MYB* genes, and purple nodes represent *LEC1* genes. Green nodes represent remaining TFs. Outside purple circles, points represent the enzymatic genes of the fatty acid biosynthesis pathway, locating on the top. Level 1 to Level 9 indicate the levels identified in the f-ordered gene co-expression network.

In fruits, a co-expression network comprising 82 TFs and 586 structural genes across five time points (F1-F5) was reconstructed with threshold values of 0.8 (positive) and -0.53 (negative) (**Figure 5A**). In leaves, a co-expression network consisting of 556 TFs and 61 structural genes across nine time series (L1-L9) was reconstructed using threshold values of 0.85 (positive) and -0.63 (negative) (**Figure 5B**). Among these networks, key fatty acid biosynthesis TF families were revealed, including bZIPs, B3, WRI, DOF, MYB and LEC1. Notably, bZIP was the most abundant TF in both fruits and leaves, with 30 and 23 members, respectively. Overall, the time-series co-expression networks provided crucial insights into the transcriptional control of fatty acid biosynthesis in both fruit and leaf development in yellowhorn.

### Potential gene regulatory network of carbon chain elongation of fatty acid biosynthesis

**Figure 6:**
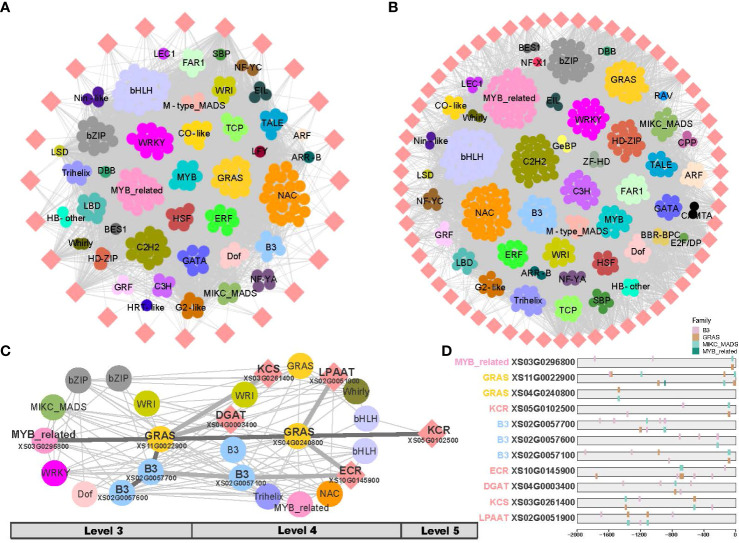
Sub-networks of very long-chain fatty acid (VLCFA) biosynthesis in fruit. **(A)** Sub-network of the phase of *de novo* fatty acid biosynthesis in fruit. **(B)** Sub-network of the phase of fatty acid elongation in fruit. **(C)** Resolved hierarchical regulation for KCR gene. **(D)** DNA binding sites detected in the 2 Kb upstream sequences of core genes and the potential regulatory elements (here TFs).

The biosynthesis of VLCFA consists of two phases: *de novo* fatty acid biosynthesis (C ≤ 18) and fatty acid elongation (C > 18) (**Figure 1B**). To analyze the two phases separately, two gene co-expression subnetworks were generated separately from fruit and leaf development, respectively (**Figures 6A, B**; **Supplementary Figure 10**). In the fruit, TF families such as bHLH, NAC, MYB_related, C2H2, GRAS, WRKY, bZIP and C3H were found in higher numbers in the networks of both phases of fatty acid biosynthesis (**Figure 6A**). In the phase of *de novo* fatty acid biosynthesis of fruit, the TF families ERF and Dof were shown a higher representation (**Figure 6A**); and in the phase of fatty acid elongation, the TFs B3 and HD-ZIP displayed a higher proportion (**Figure 6B**). In the leaf, higher numbers of TF families such as bHLH, GRAS, C3H, C2H2, WRI, HD-ZIP, and C2H2 were observed in the networks of both phases of fatty acid biosynthesis. In the phase of *de novo* fatty acid biosynthesis in leaf, the TF families B3, Dof, SBP, and bZIP showed higher proportions (**Supplementary Figure 10A**); whereas in the phase of fatty acid elongation, the TF families MYB-related, NAC, Trihelix, and WRKY had higher proportions (**Supplementary Figure 10B**).

KCS, KCR, and ECR are essential elongases involved in the VLCFA biosynthesis, with KCS being the key rate-limiting enzyme. LPAAT and DGAT are also important enzymes in the VLCFA biosynthesis. However, the relationship between key enzymatic genes and regulatory genes remained unclear. In the fruit, we identified the potential first-order to third-order (Level 3 - Level 6) upstream regulatory factors by examining the co-expression network inferred from TO-GCNs (**Figure 6C**; **Supplementary Table 8**).

Our analysis revealed that the KCR gene (*XS05G0102500*) acts as a central hub gene in the putative regulatory network in fruit development (**Figure 6C**). We inferred that the KCR gene might be hierarchically regulated by MYB-related (*XS03G0296800*) as the second regulator, GRAS (*XS11G0022900*) as the intermediate second regulator, and GRAS (*XS04G0240800*) as the direct regulator. Additionally, the enzyme genes of KCS, ECR, DGAT, and LPAAT may also be hierarchically regulated by B3 or GRAS genes, thereby enhancing the efficiency of very long-chain fatty acid biosynthesis. In subsequent analyses, when results from DNA binding site prediction were added we observed the presence of binding sites for upstream regulators on these critical genes (**Figure 6D**; **Supplementary Table 9**). Through a comprehensive investigation of putative hierarchical regulation in the fatty acid biosynthesis pathway, we inferred MYB-related (*XS03G0296800*) and GRAS (*XS11G0022900*) as important upstream regulatory factors in these pathways, in fruit development.

However, such multi-layered regulatory sub-networks were not found in leaf development.

## Discussion

To elucidate the expression pattern of enzymatic genes in the fatty acid biosynthesis pathway and to infer the potential key structural genes and the related regulatory genes, we investigated the landscape transcriptomes in fruit and leaf development with a time-serious design. A total of 137 genes encoding enzymes involved in fatty acid biosynthesis were identified in the yellowhorn genome. Most of these genes were differentially expressed during fruit development, with higher expression levels detected during the fruit expansion stage (F2-F5) compared to early (F1) and late (F6) developmental phases. The expression pattern likely supported the increased demand for fatty acids during rapid fruit growth (**Figure 1**). In contrast, more variable expression profiles were observed for biosynthetic genes in developing leaves, suggesting distinct regulatory mechanisms in these tissues. Moreover, several fatty acid synthase genes, including those encoding KAS I, KAR, KCS, and KCR, were consistently highly expressed in both fruits and leaves. These genes may play rate-limiting roles in governing carbon flux through the pathway. Interestingly, some genes showed tissue-specific patterns, such as genes (*XS07G0210900*, *XS03G0322000* and *XS03G0321200*) encoding SAD which were induced specifically during fruit development.

At the same time, we found that the distribution of these enzyme-encoding genes on chromosomes was not uniform, biosynthesis gene clusters were identified on multiple chromosomes. In the present study, the genes in these clusters are farther apart from each other, some are spanning genomic range of several Mb. After a references review, we found it is still hard to say how large in genomic range a gene cluster could be. Also, we can see one gene-gene distance could occupy 1.8 Mb (distance between *XS01G0315800*-*XS01G0341200* in one gene cluster found in Chr01). The longer gene-gene distance may be specific to the focal species in our study, and thus we cannot simply reject a potential true gene cluster only by the genomic range it is spanning. So, we would like to remain our identified gene clusters. In Arabidopsis, we do find relatively small and compact gene clusters, with the largest gene cluster being several hundred kilobases in size (Nutzmann and Osbourn, 2014). However, there are also some reported gene clusters that are less tightly linked (Nutzmann et al., 2016). Furthermore, the length of certain genes themselves occupies a considerable range, spanning up to 12 kb for a single gene (*XS07G0064100* from Chr07). Notably, the gene cluster on Chr07 contained most of the enzymatic genes during the phase of fatty acid elongation, which likely improved the synthesis efficiency of VLCFAs to some extent (**Figure 2B**). Overall, the specialized expression profiles of individual genes coupled with coordinated transcription of clustered genes may provide potential clues for directing fatty acid biosynthesis in fruit and leaf.

Oleosins play crucial roles in stabilizing oil bodies where VLCFAs are stored as triacylglycerols (TAGs) in seeds and fruits. Characterizing oleosin diversity and expression patterns informs how oil accumulation is controlled. Phylogenetic analysis identified four *oleosin* genes in yellowhorn, which clustered into three conserved oleosin gene families (SL, SH and U clades) known to be involved in oil body stabilization (**Figure 3**). The *Oleosin* genes from the three clades displayed fruit-specific expression profiles, consistent with their specialized roles in regulating oil accumulation during fruit maturation. It has been reported that five oleosin genes are specifically expressed in mature seeds of *Arabidopsis thaliana* (Kim et al., 2002). Variation in gene structure and conserved motifs between the oleosin subfamilies likely contributed to their functional diversification. Overall, elucidating the *Oleosin* gene families in yellowhorn provides foundational insights into the VLCFAs and yellowhorn oil accumulation.

Through comparing the DEGs between fruit and leaf, we identified 2,083 and 3,575 specifically expressed genes, respectively. We expected more GO terms related to VLCFA biosynthesis to be enriched in fruit. Interestingly, growth and development related genes were predominantly enriched in fruit, while fatty acid synthesis relevant GO terms, glycerolipid biosynthetic process and lipoprotein metabolic process, were enriched in leaf. Here, it is worth noting that leaves of yellowhorn have a prominent cuticular wax layer, which helps protect against environmental biotic and abiotic stresses (Zhao et al., 2021). In addition, we speculated that leaves may be the actual site of VLCFA synthesis, while fruits simply accumulate the oils.

In recent years, TFs including B3, Dof, HD-ZIP, AP2, and CBP families have been favored for their ability to regulate fatty acid biosynthesis, which can increase the yield and quality of vegetable oils. Studies have shown that these TFs play important roles in the accumulation of VLCFAs in plant seeds and fruits. For example, the B3 transcription factor FUS3 is involved in the regulation of VLCFA accumulation in Arabidopsis seeds (Roscoe et al., 2015). The B3 transcription factor KLUH is associated with lipid metabolism and organ growth in tomato (Li et al., 2021). These TFs form a metabolic network rather than acting alone. Time-ordered transcriptome data enabled construction of developmental stage-specific gene co-expression networks in fruit and leaf. The TF families bZIP, B3, and WRI were abundant though unevenly distributed across different levels in both fruit and leaf. We further extracted subnetworks based on the phase of *de novo* fatty acid biosynthesis and fatty acid elongation. In fruit, more TFs ERF and Dof were involved in *de novo* synthesis compared to elongation (**Figure 6A**). More TFs B3 and HD-ZIP were active during the phase of elongation compared to *de novo* fatty acid synthesis (**Supplementary Figure 10A**). Some members of these TF families play important roles in different stages of plant fruit development (Baud et al., 2008; Ye et al., 2021; Li et al., 2022), e.g., the soybean Dof-type transcription factor genes have an important role in the regulation of soybean seed lipid content (Wang et al., 2007). In leaves, more TFs B3, Dof, SBP and bZIP are involved in the phase of *de novo* synthesis compared to elongation. And TF families MYB-related, NAC, Trihelix and WRKY were active during the phase of elongation (**Supplementary Figure 10B**). Furthermore, by integrating hierarchical clustering analysis and DNA binding site prediction, we inferred that MYB_related (*XS03G0296800*) and B3 (*XS02G0057600*) transcription factors act as top regulators that can control the conversion of carbon flux into fatty acids.

In summary, we investigated the transcriptional regulation of genes involved in fatty acid biosynthesis during fruit and leaf development in yellowhorn. Our results provide novel insights into key genes and transcriptional regulators governing fatty acid production in this species. This work lays the foundation for future efforts to optimize oil content and fatty acid composition in yellowhorn through elucidation of the transcriptional networks directing carbon flux into oils. The gene expression patterns and putative regulatory relationships identified here will inform metabolic engineering and molecular breeding approaches to tailor oil synthesis to meet biofuel and bioproduct needs. Overall, this study advances the knowledge of the genomics underpinning oil metabolism in an important biofuel crop.

## Data availability statement

The datasets presented in this study can be found in online repositories. The names of the repository/repositories and accession number(s) can be found below: https://www.ncbi.nlm.nih.gov/, PRJNA694500.

## Author contributions

T-LS: Writing – original draft. H-YM: Writing – original draft. XW: Writing – review & editing. HL: Writing – review & editing. X-MY: Writing – review & editing. X-CT: Writing – review & editing. Z-CL: Writing – review & editing. Y-TB: Writing – review & editing. Z-YC: Writing – review & editing. S-WZ: Writing – review & editing. QX: Writing – review & editing. K-HJ: Writing – review & editing. SN: Writing – review & editing. WG: Writing – review & editing. J-FM: Writing – review & editing.
