# Supplementary material for: Differential gene expression and potential regulatory network of fatty acid biosynthesis during fruit and leaf development in yellowhorn (Xanthoceras sorbifolium), an oil-producing tree with significant deployment values
Source: Front Plant Sci. 2024 Jan 19;14:1297817. doi: 10.3389/fpls.2023.1297817 (PMC10834690; doi:10.3389/fpls.2023.1297817)
Supplement: Supplementary file 1 [file DataSheet_1.docx]

**Supplementary Figures**


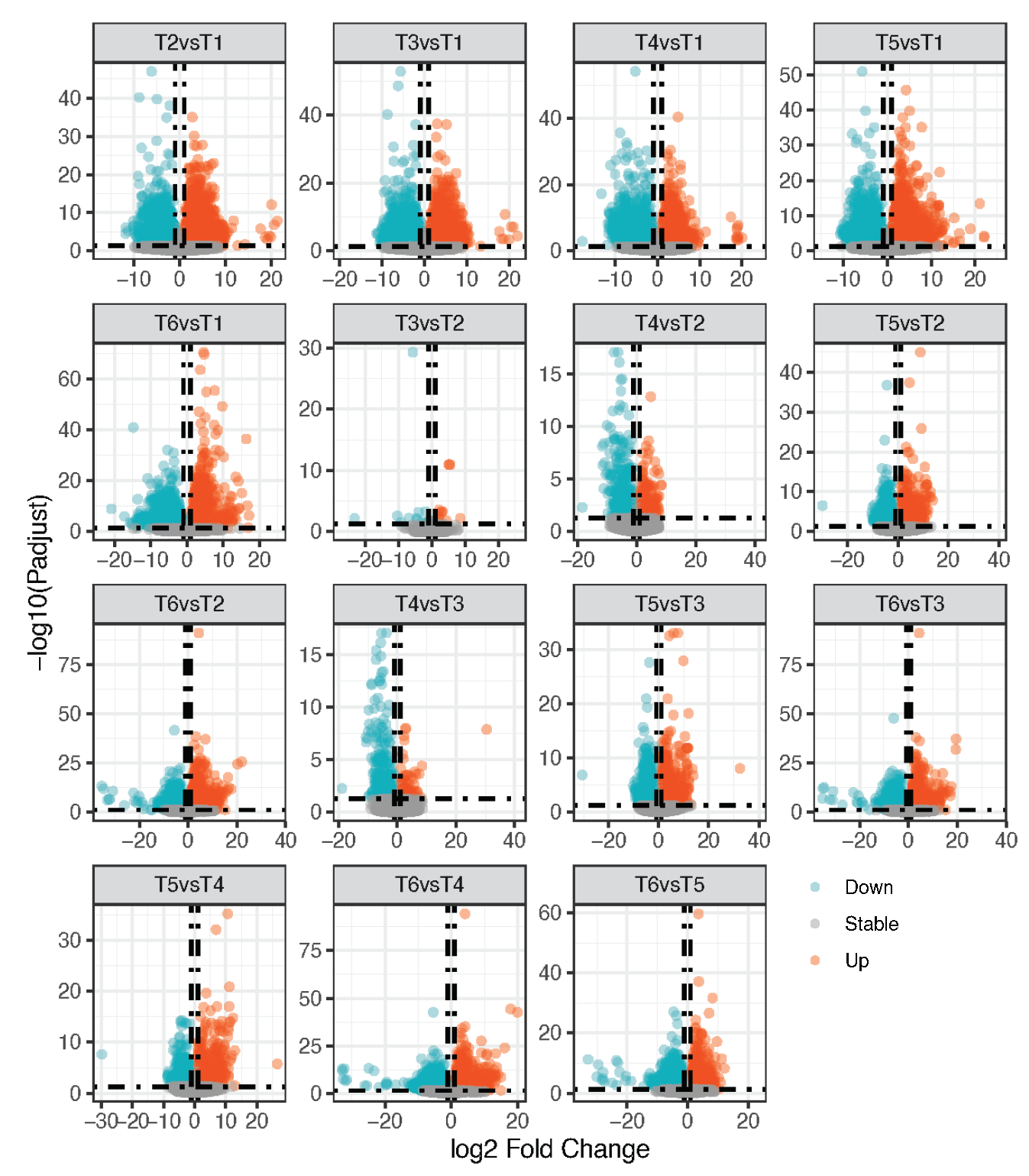


**Supplementary Figure 1. Volcano plot of gene expression at six different developmental stages (F1–F6) in fruit.** Red dots indicate genes with significantly increased expression (upregulated). Blue dots indicate genes with significantly decreased expression (downregulated). Gray dots are for non-significant expression.


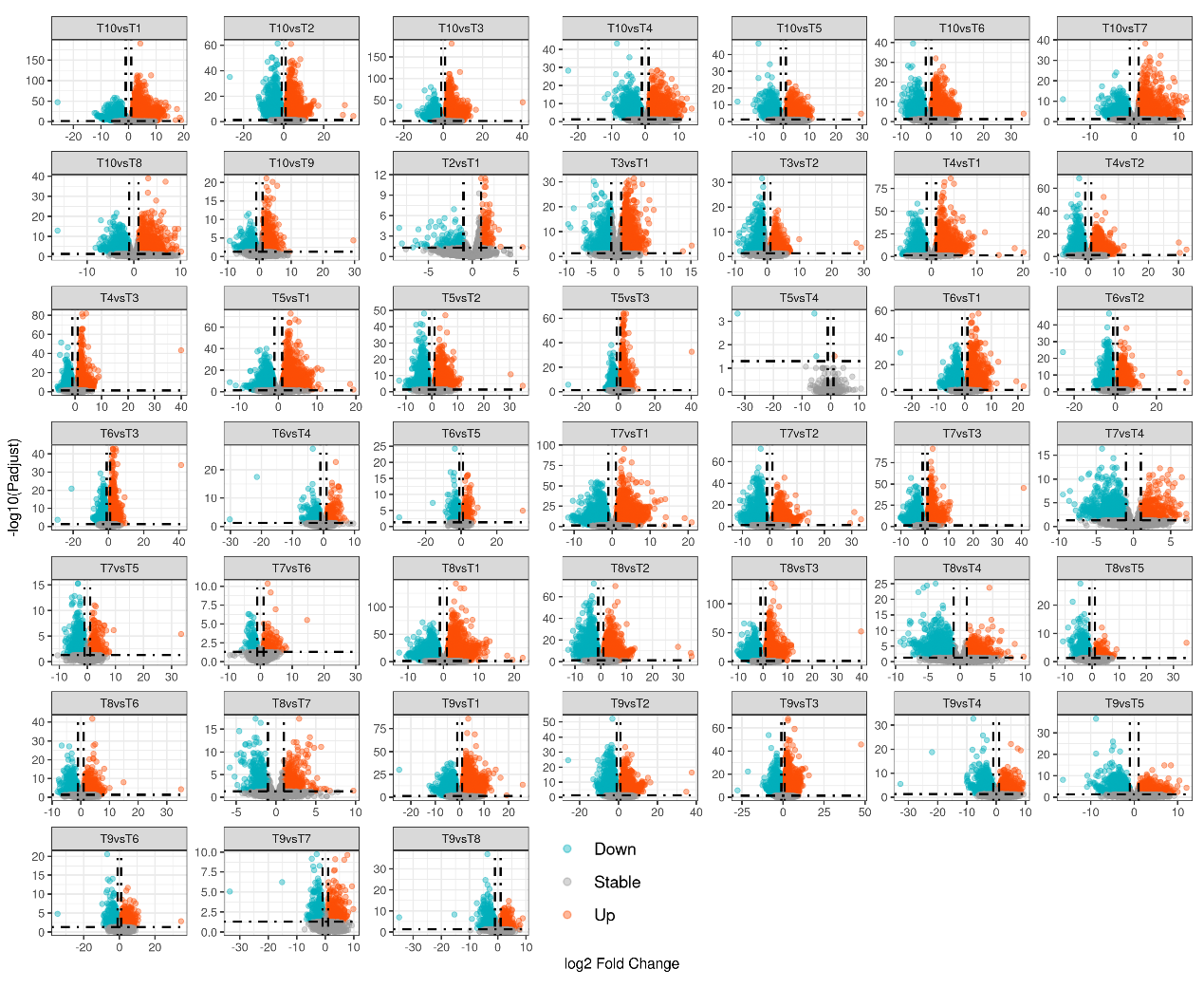


**Supplementary Figure 2. Volcano plot of gene expression at ten different developmental stages (L1–L10) in leaf.** Red dots indicate genes with significantly increased expression (upregulated). Blue dots indicate genes with significantly decreased expression (downregulated). Gray dots are for non-significant expression.


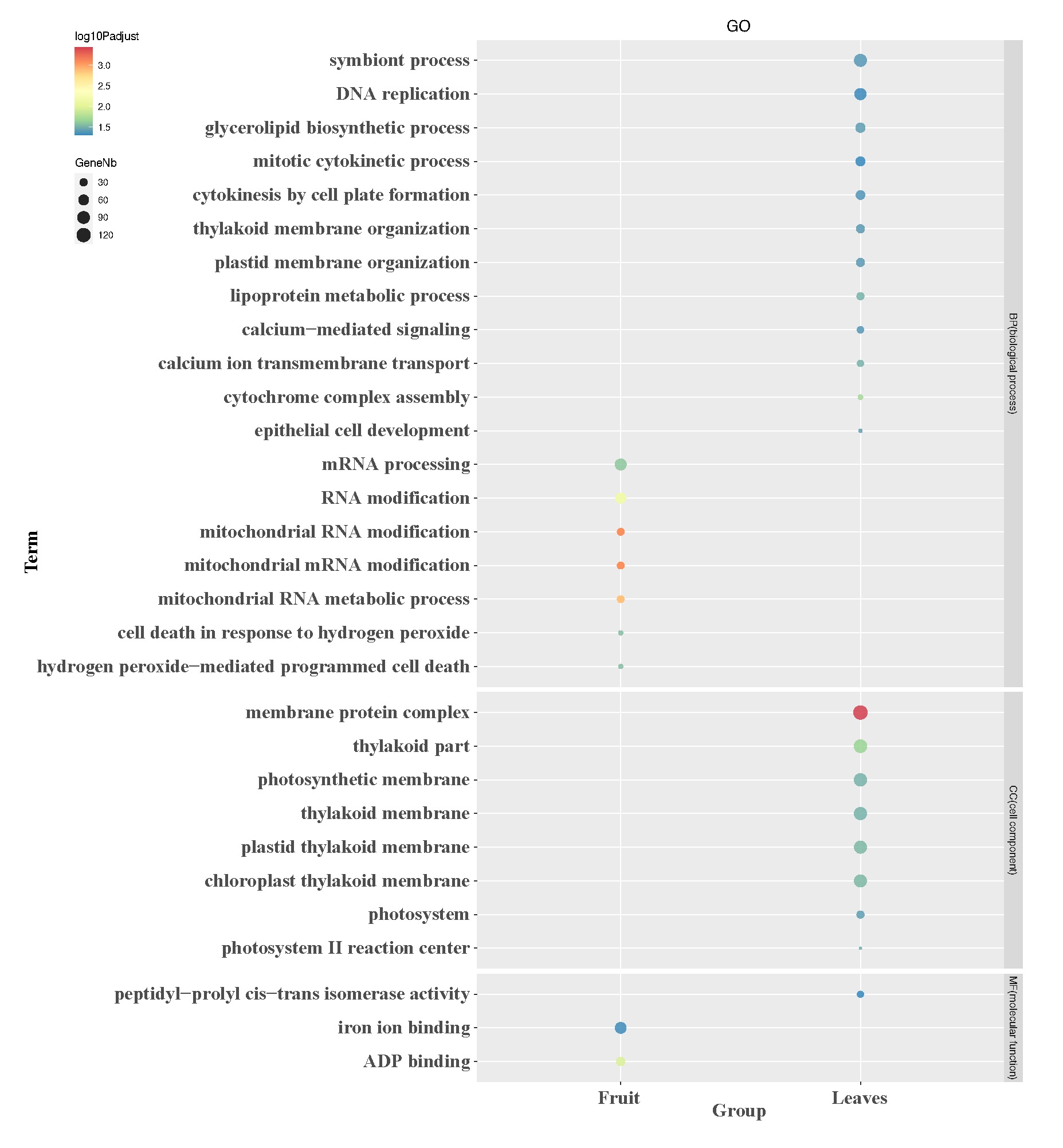


**Supplementary Figure 3. Gene Ontology (GO) enrichment analysis for specifically expressed differentially expressed genes (DEGs) in fruit and leaf.** The enriched GO terms with corrected *P* value < 0.05 are presented. The color of circles represents the statistical significance of the enriched GO terms. The size of the circles represents the number of genes in a GO term. ‘*P* adjust’ is the Benjamini-Hochberg false discovery rate (FDR) adjusted *P* value.


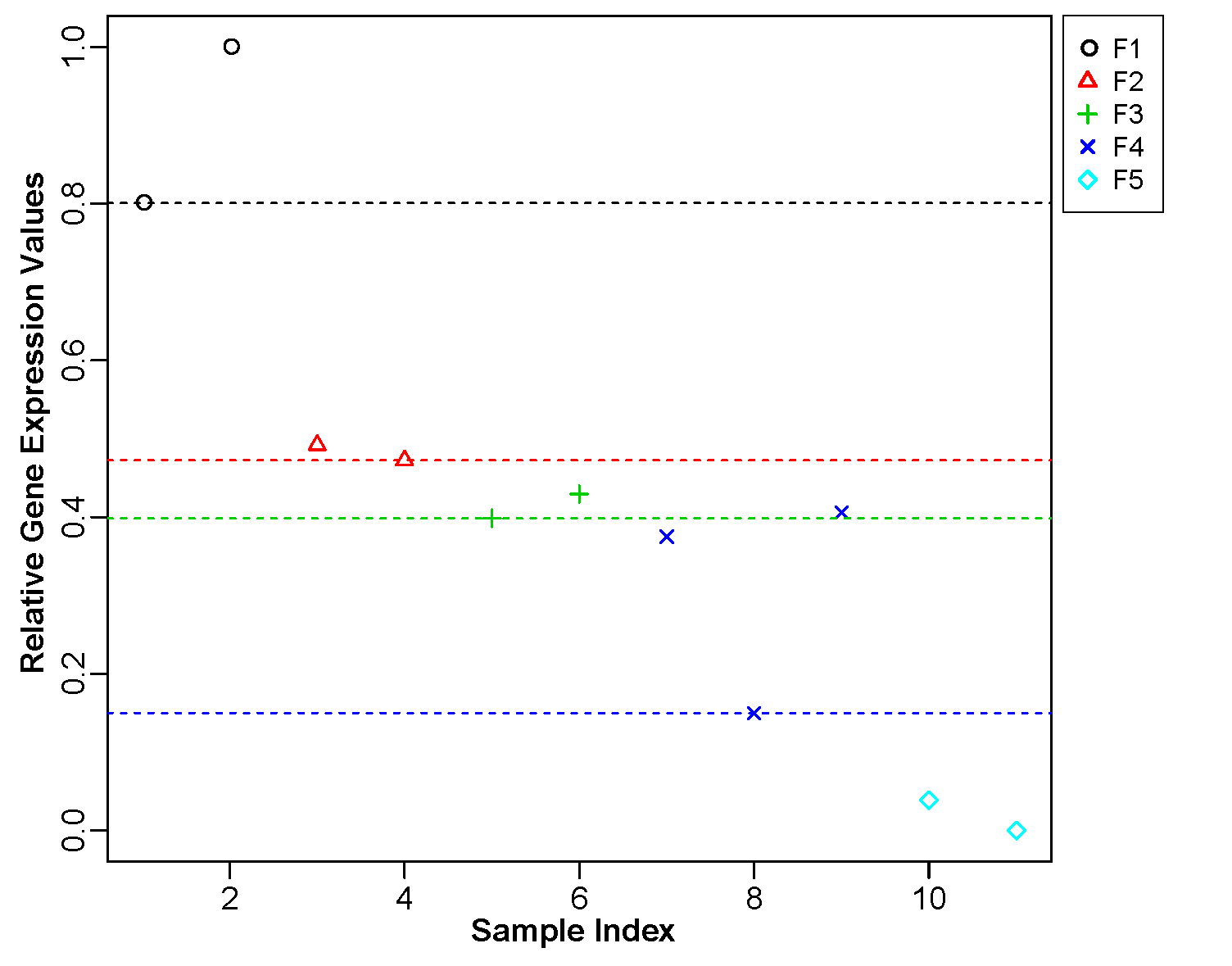


**Supplementary Figure 4. Relative expression values of *XS01G0336800* which was used as the bait gene in generation of the time-ordered gene co-expression network (TO-GCN) in fruit.** The same symbol represents biological replications from the same developmental stage.


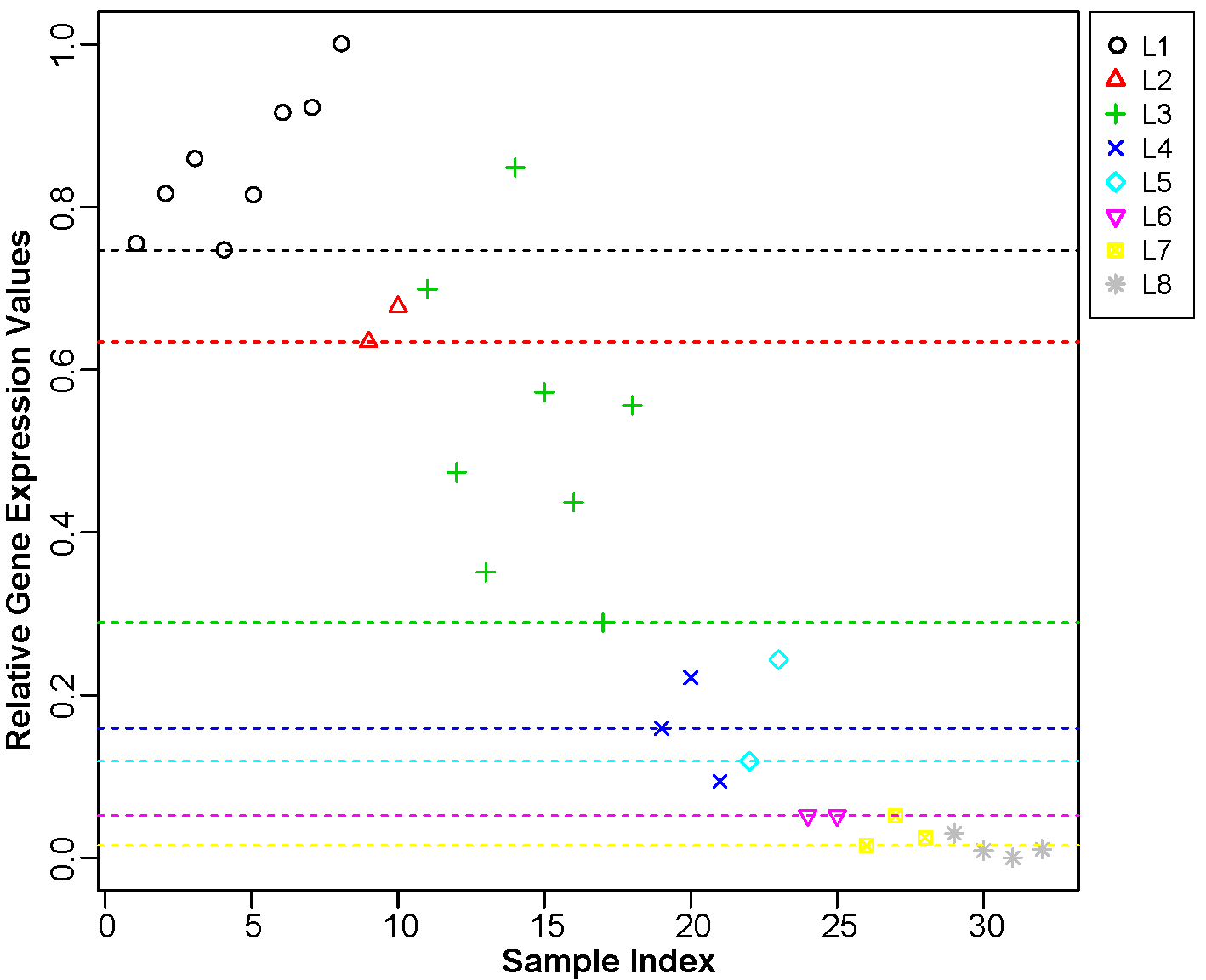


**Supplementary Figure 5. Relative expression values of *XS03G0212300* which was used as the bait gene in generation of the time-ordered gene co-expression network (TO-GCN) in leaf.** The same symbol represents biological repetitions from the same developmental stage.


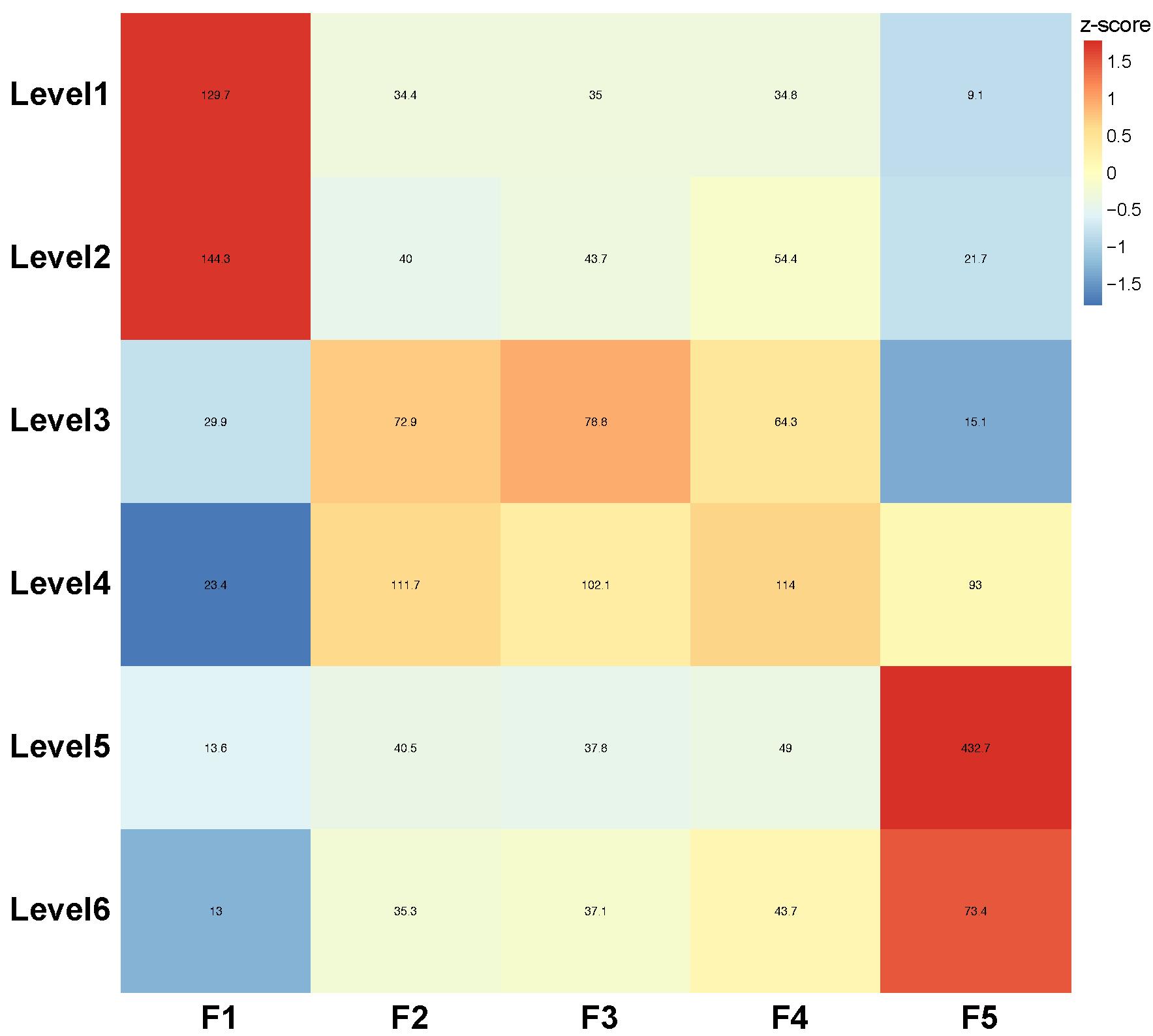


**Supplementary Figure 6. The heatmap of average normalized TPMs for each developmental stage of fruit at each level identified in the time-ordered gene co-expression network (TO-GCN).** F1-F5 indicate five stages of fruit development. Low to high expression is shown by an adjustment from blue to red.


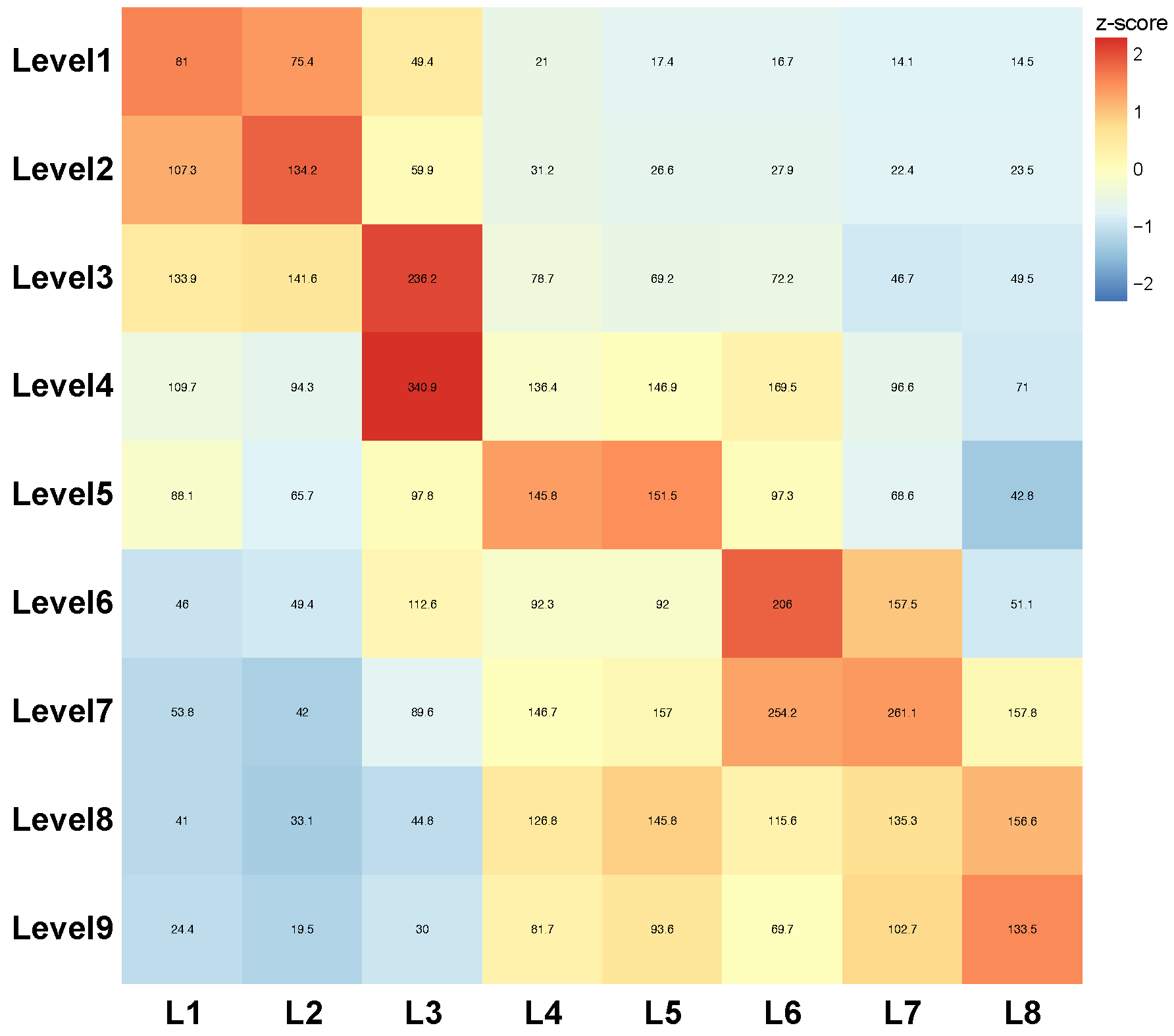


**Supplementary Figure 7. The heatmap of average normalized TPMs for each developmental stage of leaf at each level identified in the time-ordered gene co-expression network (TO-GCN).** L1–L8 indicate eight stages of leaf development. Low to high expression is shown by an adjustment from blue to red.


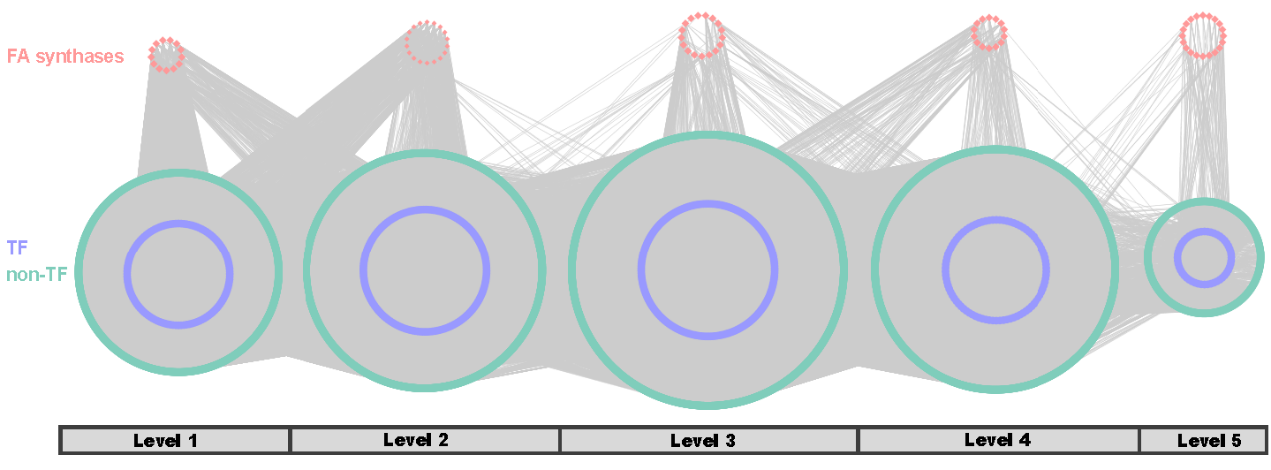


**Supplementary Figure 8. Predicted regulatory network and the connection among fatty acid synthases and regulatory genes (non-TFs and TFs) involved in very long-chain fatty acid (VCLFA) biosynthesis pathway in fruit.** Green nodes represent TFs. Pink points (outside green circles) represent the enzymatic genes of the fatty acid biosynthesis pathway. Purple nodes (inside green circles) represent non-TF genes. Level 1-5 indicate the levels identified in the TO-GCN network.


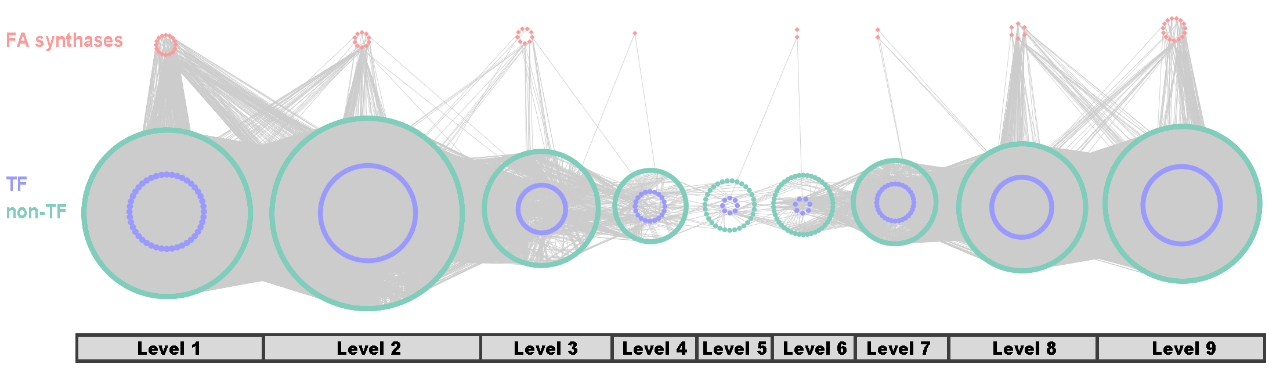


**Supplementary Figure 9.** **Predicted regulatory network and the connection among fatty acid synthases and regulatory genes (non-TFs and TFs) involved in very long-chain fatty acid (VLCFA) biosynthesis pathway in leaf.** Green nodes represent TFs. Pink points (outside green circles) represent the enzymatic genes of the fatty acid biosynthesis pathway. Purple nodes (inside green circles) represent non-TF genes. Level 1-9 indicate the levels identified in the TO-GCN network.


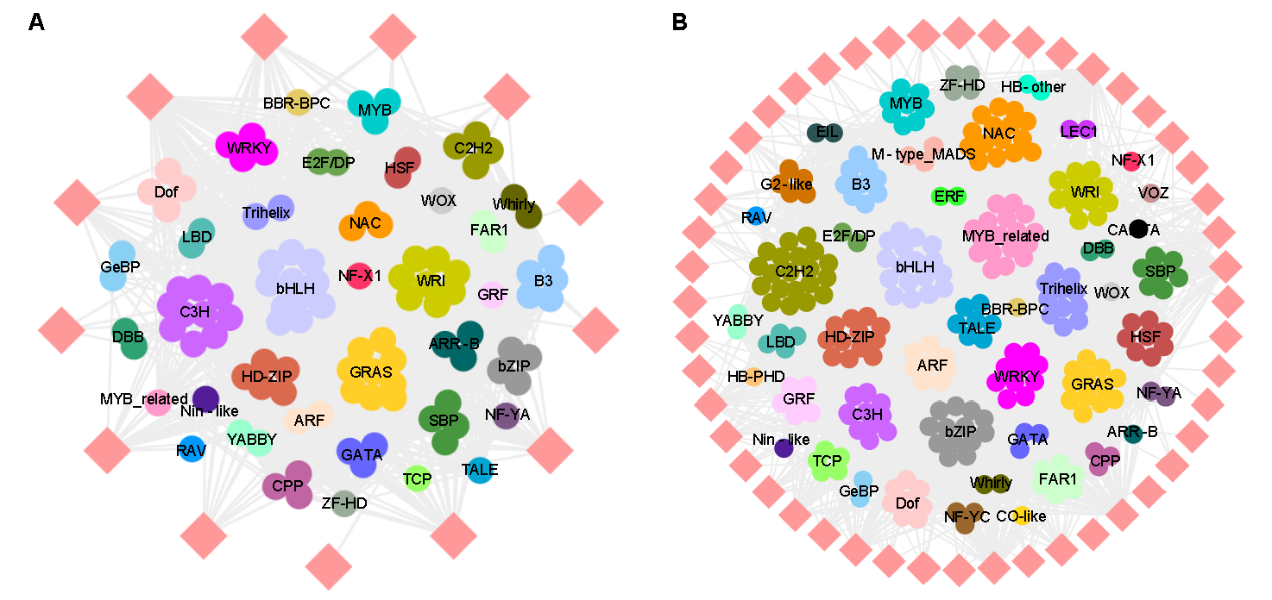


**Supplementary Figure 10. Sub-network related to *de novo* and elongation phases of fatty acid biosynthesis in fruit. A** Sub-network related to the phase of *de novo* fatty acid biosynthesis in leaf. **B** Sub-network of the phase of fatty acid elongation in leaf.
